# Supplementary material for: Transient Autoreactive PF4 and Antiphospholipid Antibodies in COVID-19 Vaccine Recipients
Source: Vaccines (Basel). 2023 Dec 14;11(12):1851. doi: 10.3390/vaccines11121851 (PMC10747426; doi:10.3390/vaccines11121851)
Supplement: Supplementary file 1 [file vaccines-11-01851-s001.zip › vaccines-2692380-supplementary.pdf]

Supplementary Materials

Text S1. Direct acyclic graph and assumptions

The assumptions underlying the cross−sectional analysis in the main text are summarized in e−Figure 1. In this analysis, we estimated the correlations between PF4 antibody (aPF4) levels and various covariates, including sex, age at vaccination, prior COVID−19 infection status, type of vaccination and study center. We assumed that each correlation is confounded by a distinct set of predefined covariates. As a result, we performed multiple multivariable analyses, using different covariates for each correlation.

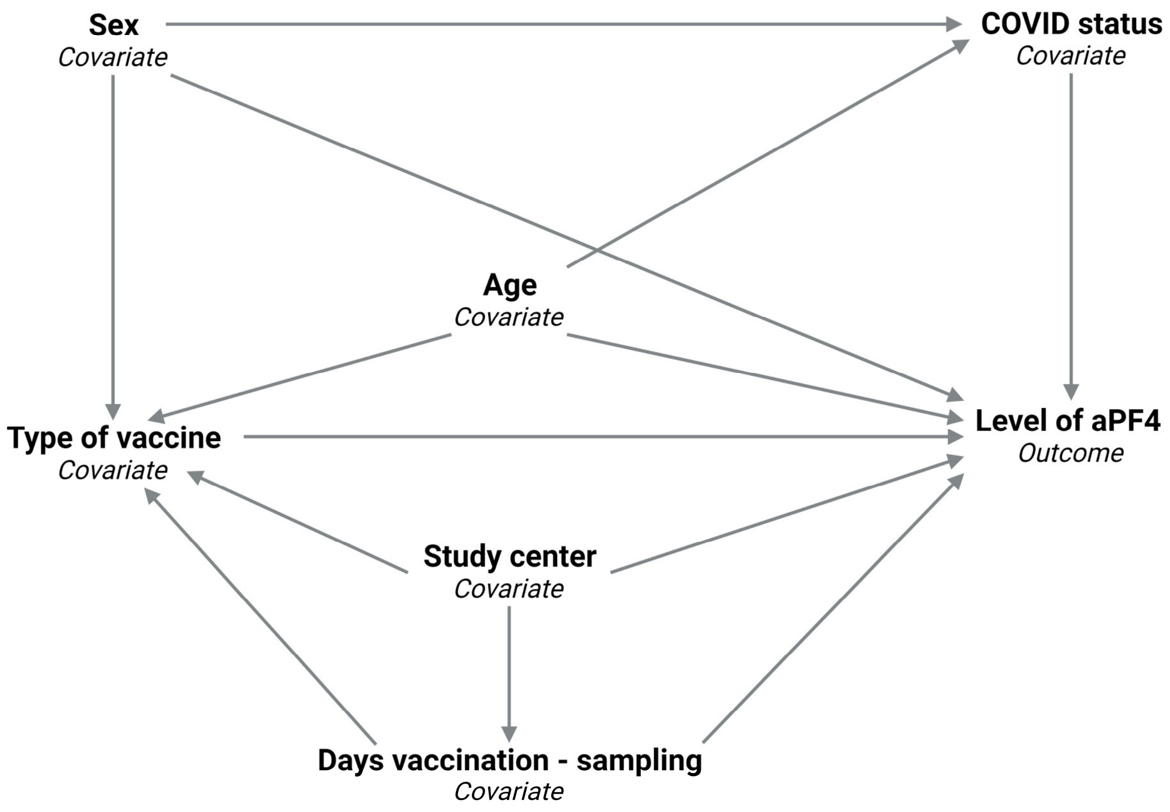

Figure S1. The correlation between the different covariates and the level of PF4 antibodies. The correlation between level of aPF4 and the different covariations and the confounders and mediators of these correlations illustrated in a directed acyclic graph. The arrows indicate the direction of the relationship.

Table S1 Correlations between aPF4 and aPL antibodies in every vaccine recipient

		At time point T1				At time point T2				At time point T3			
		aPF4 IgG (O.D units)	aβ2GP total Ig (U/mL)	aCL IgM (U/mL)	aCL IgG (U/mL)	aPF4 IgG (O.D units)	aβ2GP total Ig (U/mL)	aCL IgM (U/mL)	aCL IgG (U/mL)	aPF4 IgG (O.D units)	aβ2GP total Ig (U/mL)	aCL IgM (U/mL)	aCL IgG (U/mL)
T1	aPF4 IgG (O.D units)		−0.03	−0.06	0.11	0.81**	−0.05	0.00	0.14	0.81**	0.05	0.04	0.22
	aβ2GP total Ig (U/mL)	−0.03		0.53**	0.54**	−0.01	0.86**	0.47**	0.49**	−0.04	0.87**	0.41**	0.42**
	aCL IgM (U/mL)	−0.06	0.53**		0.39**	−0.09	0.55**	0.88**	0.37**	−0.14	0.36**	0.87**	0.30**
	aCL IgG (U/mL)	0.11	0.54**	0.39**		0.14	0.44**	0.35**	0.75**	0.06	0.40**	0.32**	0.65**
T2	aPF4 IgG (O.D units)	0.81**	−0.01	−0.09	0.14		−0.05	−0.07	0.17	0.86**	−0.01	−0.09	0.25
	aβ2GP total Ig (U/mL)	−0.05	0.86**	0.55**	0.44**	−0.05		0.51**	0.54**	−0.11	0.86**	0.47**	0.49**
	aCL IgM (U/mL)	0.00	0.47**	0.88**	0.35**	−0.07	0.51**		0.40**	−0.15	0.35**	0.86**	0.33**
	aCL IgG (U/mL)	0.14	0.49**	0.37**	0.75**	0.17*	0.54**	0.40**		0.17	0.34**	0.38**	0.78**
T3	aPF4 IgG (O.D units)	0.81**	−0.04	−0.14	0.06	0.86**	−0.11	−0.15	0.17		−0.06	−0.07	0.20*
	aβ2GP total Ig (U/mL)	0.05	0.87**	0.36**	0.40**	−0.01	0.86**	0.35**	0.34**	−0.06		0.40**	0.47**
	aCL IgM (U/mL)	0.04	0.41**	0.87**	0.32**	−0.08	0.47**	0.86**	0.38**	−0.07	0.40**		0.47**
	aCL IgG (U/mL)	0.22*	0.42**	0.30**	0.65**	0.24**	0.49**	0.33**	0.78**	0.20*	0.47**	0.47**	

Abbreviations: aPF4: anti−Platelet Factor 4; O.D: Optical Density; IgG: Immunoglobulin G; aβ2GP: anti−beta2−Glycoprotein; Ig; Immunoglobuline; aCL: anti−CardioLipin; IgM: Immunoglobulin M. Numbers indicate Spearman’s rank correlation coefficient with continuity correction. *p < 0.05, **p < 0.01

Table S2 Correlations between aPF4 and aPL antibodies in mRNA−based vaccine recipients

		At time point T1				At time point T2				At time point T3			
		aPF4 IgG (O.D units)	aβ2GP total Ig (U/mL)	aCL IgM (U/mL)	aCL IgG (U/mL)	aPF4 IgG (O.D units)	aβ2GP total Ig (U/mL)	aCL IgM (U/mL)	aCL IgG (U/mL)	aPF4 IgG (O.D units)	aβ2GP total Ig (U/mL)	aCL IgM (U/mL)	aCL IgG (U/mL)
T1	aPF4 IgG (O.D units)		0.14	0.03	0.02	0.86**	0.05	0.08	0.05	0.81**	0.09	0.11	0.11
	aβ2GP total Ig (U/mL)	0.14		0.40**	0.50**	0.10	0.92**	0.36**	0.49**	0.10	0.90**	0.37**	0.63**
	aCL IgM (U/mL)	0.03	0.40**		0.36**	−0.03	0.41**	0.93**	0.32**	−0.02	0.36**	0.87**	0.43**
	aCL IgG (U/mL)	0.02	0.50**	0.36**		0.04	0.40**	0.36**	0.79**	0.01	0.43**	0.39**	0.75**
T2	aPF4 IgG (O.D units)	0.86**	0.10	−0.03	0.04		0.01	−0.04	0.02	0.84**	0.00	−0.05	0.06
	aβ2GP total Ig (U/mL)	0.05	0.92**	0.41**	0.40**	0.01		0.40**	0.52**	−0.01	0.88**	0.38**	0.62**
	aCL IgM (U/mL)	0.08	0.36**	0.93**	0.36**	−0.04	0.40**		0.40**	−0.05	0.36**	0.89**	0.47**
	aCL IgG (U/mL)	0.05	0.49**	0.32**	0.79**	0.02	0.52**	0.40**		0.02	0.43**	0.46**	0.81**
T3	aPF4 IgG (O.D units)	0.81**	0.10	−0.02	0.01	0.84**	−0.01	−0.04	0.02		0.01	0.05	0.07

	aβ2GP total Ig (U/mL)	0.09	0.92**	0.36**	0.43**	0.00	0.88**	0.36**	0.43**	0.01		0.37**	0.57**
	aCL IgM (U/mL)	0.11	0.36**	0.87**	0.39**	−0.05	0.38**	0.89**	0.46**	0.05	0.37**		0.51**
	aCL IgG (U/mL)	0.11	0.63**	0.43**	0.75**	0.06	0.62**	0.47**	0.81**	0.07	0.57**	0.51**	

Abbreviations: aPF4: anti−Platelet Factor 4; O.D: Optical Density; IgG: Immunoglobulin G; aβ2GP: anti−beta2−Glycoprotein; Ig; Immunoglobuline; aCL: anti−CardioLipin; IgM: Immunoglobulin M. Numbers indicate Spearman's rank correlation coefficient with continuity correction. *p < 0.05, **p < 0.01

Table S3 Correlations between aPF4 and aPL antibodies in rAV−based vaccine recipients

		At time point T1				At time point T2				At time point T3			
		aPF4 IgG (O.D units)	aβ2GP total Ig (U/mL)	aCL IgM (U/mL)	aCL IgG (U/mL)	aPF4 IgG (O.D units)	aβ2GP total Ig (U/mL)	aCL IgM (U/mL)	aCL IgG (U/mL)	aPF4 IgG (O.D units)	aβ2GP total Ig (U/mL)	aCL IgM (U/mL)	aCL IgG (U/mL)
T1	aPF4 IgG (O.D units)		−0.13	−0.14	0.22	0.76**	−0.14	−0.10	0.26	0.86**	−0.12	−0.09	0.36
	aβ2GP total Ig (U/mL)	−0.13		0.61**	0.55**	−0.13	0.79**	0.59**	0.46**	−0.22	0.83**	0.45**	0.04
	aCL IgM (U/mL)	−0.14	0.61**		0.41**	−0.15	0.68**	0.83**	0.44**	−0.25	0.40**	0.84**	0.09
	aCL IgG (U/mL)	0.22	0.55**	0.41**		0.20	0.48**	0.36**	0.75**	0.17	0.34*	0.17	0.48**
T2	aPF4 IgG (O.D units)	0.76**	−0.13	−0.15	0.20		−0.13	−0.10	0.26*	0.91**	−0.06	−0.10	0.51**
	aβ2GP total Ig (U/mL)	−0.14	0.79**	0.68**	0.48**	−0.13		0.64**	0.57**	−0.30*	0.83**	0.58**	0.24
	aCL IgM	−0.10	0.59**	0.83**	0.36**	−0.09	0.64**		0.47**	−0.28	0.35*	0.77*	0.08

	(U/mL)												
	aCL IgG (U/mL)	0.26*	0.46**	0.44**	0.75**	0.26*	0.57**	0.47**		0.37*	0.21	0.29	0.72**
T3	aPF4 IgG (O.D units)	0.86**	−0.22	−0.25	0.17	0.91**	−0.29*	−0.28	0.37*		−0.16	−0.14	0.39*
	aβ2GP total Ig (U/mL)	−0.12	0.83**	0.40**	0.34*	−0.06	0.83**	0.35**	0.21	−0.16		0.42*	0.29
	aCL IgM (U/mL)	−0.09	0.45**	0.84**	0.17	−0.10	0.58**	0.77**	0.29	−0.13	0.41*		0.42*
	aCL IgG (U/mL)	0.36*	0.04	0.09	0.48**	0.51**	0.24	0.07	0.72**	0.39*	0.29	0.42*	

Abbreviations: aPF4: anti−Platelet Factor 4; O.D: Optical Density; IgG: Immunoglobulin G; aβ2GP: anti−beta2−Glycoprotein; Ig; Immunoglobuline; aCL: anti−CardioLipin; IgM: Immunoglobulin M. Numbers indicate Spearman's rank correlation coefficient with continuity correction. *$p < 0.05$, **$p < 0.01$
